# Sensing the Inside Out: An Embodied Perspective on Digital Animation Through Motion Capture and Wearables

**DOI:** 10.3390/s25072314

**Published:** 2025-04-05

**Authors:** Katerina El-Raheb, Lori Kougioumtzian, Vilelmini Kalampratsidou, Anastasios Theodoropoulos, Panagiotis Kyriakoulakos, Spyros Vosinakis

**Affiliations:** Department of Performing and Digital Arts, University of the Peloponnese 1, 211 00 Nafplio, Greece

**Keywords:** digital animation, biosignals, sensors, motion capture, nonverbal communication, character design

## Abstract

Over the last few decades, digital technology has played an important role in innovating the pipeline, techniques, and approaches for creating animation. Sensors for motion capture not only enabled the incorporation of physical human movement in all its precision and expressivity but also created a field of collaboration between the digital and performing arts. Moreover, it has challenged the boundaries of cinematography, animation, and live action. In addition, wearable technology can capture biosignals such as heart rate and galvanic skin response that act as indicators of the emotional state of the performer. Such metrics can be used as metaphors to visualise (or sonify) the internal reactions and bodily sensations of the designed animated character. In this work, we propose a framework for incorporating the role of the performer in digital character animation as a real-time designer of the character’s affect, expression, and personality. Within this embodied perspective, sensors that capture the performer’s movement and biosignals are viewed as the means to build the nonverbal personality traits, cues, and signals of the animated character and their narrative. To do so, following a review of the state of the art and relevant literature, we provide a detailed description of what constitute nonverbal personality traits and expression in animation, social psychology, and the performing arts, and we propose a workflow of methodological and technological toolstowardsan embodied perspective for digital animation.

## 1. Introduction

Over the past few decades, digital animation has evolved significantly, expanding in previously unknown waters with the integration of motion capture and biosignal-driven technologies. While traditional animation methods rely on predefined emotion classification frameworks, recent developments in artificial intelligence and sensor-based technologies offer new possibilities for creating dynamic real-time character expressions based on movement and physiological responses. However, a significant gap remains in how embodied interaction and sensor-based data can be effectively operationalised to drive animation workflows, due to the lack of standardised methodologies or integration frameworks.

Embodiment in computing and human–computer interaction refers mainly to the interaction with machines using the whole body and communicating through nonverbal expressions and gestures. In eXtended Reality and games research, the Sense of Embodiment [1] refers to not only controlling a digital body directly with one’s own body (hands, locomotion, etc.) but also creating the illusion that this body feels like one’s own body. Studies have shown that the way this digital body (avatar) looks in such experiences is capable of even altering the movement behavior of the user (Proteus effect) [2,3]. Embodiment in philosophy has been established for many years since Merleau Ponty [4] questioned the Cartesian dichotomy between the mind and the body. Since then, embodiment has been studied and developed in many fields ranging from philosophy [5,6] to linguistics [7], psychology [8], cognitive science [9], and human–computer interaction [10,11].

Embodiment approaches the phenomenon/process of not just having a body but that someone (or an agent) is aware of its own body, its shape, appearance, morphology, articulation, function, and ability to sense the environment through it, perceive external stimuli, and translate them into meaningful information for action [12,13]. Through this lens, the body is not a machine or hardware operated by the mind but the mind is embodied and the body is thinking through its kinesthetic ability and simultaneous processing of multimodal stimuli received through its sensorial system. Additionally, embodied perception extends to the way we sense and understand internal and external sensations through our bodies, e.g., temperature, sweating, feeling cold or warm, or pain. Theories of embodied cognition support the idea that meaning-making in language happens through conceptual metaphors [14], that is, through using embodied experiences, feelings, and actions as analogies for understanding and communicating abstract concepts; e.g., the discussion was cold; the atmosphere was warm. We often also use analogies from one modality to another, for example, warm colors or strong taste. The concept of embodiment extends into digital animation and interactive media, where motion capture and movement sensors allow for a direct translation of human bodily expression into animated characters. This technological evolution marks a significant shift in animation history, reinforcing the idea that cognition and meaning-making are deeply tied to bodily experience. Contemporary animation can be categorised based on how motion and action are generated, including (a) keyframing,(b) real-time game actions, (c) puppeteering, (d) computer generation, or (e) hybrid approaches. In keyframing, animators meticulously craft motion sequences, reminiscent of traditional animation techniques, while real-time game animation introduces player-controlled movement within predefined constraints. Puppeteering [15] further bridges the gap between human movement and digital embodiment as performers physically manipulate a character in real time, simultaneously acting, dancing, and improvising within a narrative framework. In such workflows, the character’s identity emerges both through its designed morphology and the performative embodiment of the actor [16,17].

In computer-generated animation, character movement is dictated entirely by programmed rules or artificial intelligence, constructing a form of digital embodied cognition. Across all these approaches, the movement of an animated character, like that of a living being, serves multiple functions: (a) expressing internal thoughts, emotions, and moods, (b) performing everyday actions aligned with its environment, (c) reacting to other beings through social interactions, and (d) adapting behavior in response to perceived changes in its surroundings. These embodied movements highlight how digital bodies, like physical ones, engage with their world through perception, action, and meaningful interaction, reinforcing the intertwined nature of body and cognition.

In this work, we investigate an embodied perspective to character design in digital animation that emphasises nonverbal behavior. Building on the idea that embodiment is not only about ’having and controlling’ a body but also about perceiving the external world, internal sensations, and interactions with both human and non-human entities through the body, we highlight the significant role sensors play in embodied character design. This study also explores how biosignal-driven animation can enhance nonverbal personality traits in digital characters. Specifically, we propose a dynamic-sensor-based framework to character design that extends beyond rigid emotion classification, allowing for real-time personality-driven animation. By integrating movement analysis techniques with biosignal data, we establish a workflow that translates physiological and motion cues into expressive character behaviors. The proposed methodology aims to bridge the gap between cognitive science, human–computer interaction, and animation technology, offering a structured approach to achieving real-time personality-driven animation. To structure this investigation, we examine this concept through multiple lenses within the contexts of different fields, providing a comprehensive literature review towards creating a unified embodied animation workflow.

The structure of the paper is outlined as follows: Section 2 examines the embodied perspective in animation as it has been perceived and utilised in previous and current works. Section 3 provides an introduction to embodied character design through examining frameworks and definitions on embodied behavior and nonverbal communication. Section 4 examines character personality as a response mechanism and how it can inform character animation. Regarding more technical aspects, Section 5 presents an overview of the state of the art of sensor-based animation, while Section 6 explores methods of integrating sensors in animation workflow. Finally, Section 7 presents the proposed workflow derived from this work and discusses key challenges and limitations, while Section 8 concludes the paper.

## 2. The Embodied Perspective in Animation

**Figure 1:**
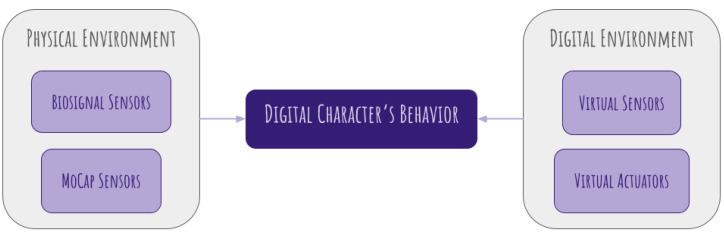
Simplified model of how physical and digital environments interact to shape the behavior of a digital character.

Before examining the embodied perspective in animation, it is useful to outline the core framework of this study. The following diagram presents a simplified model of how physical and digital environments interact to shape the behavior of a digital character (Figure 1). The subsequent sections will analyze each of these elements in detail, leading to a refined workflow.

Cognitive philosopher Andy Clark (2007), writing before the rapid technological advancements in AI, wearables, and motion capture, described three grades of embodiment: mere, modest, and profound embodiment [5]. He explains, “A ‘merely embodied’ creature or robot would be one equipped with a body and sensors, able to engage in closed-loop interactions with its world, but for whom the body was nothing but a means to implement solutions arrived at by pure reason… Profoundly embodied agents, on the other hand, have boundaries and components that are forever negotiable, where body, thinking, and sensing are interwoven flexibly (and repeatedly) within the fabric of situated, intentional action” [5]. In the field of animation, the concept of embodiment is defined as the perception of bodily movement that goes beyond its physiological nature, viewing it as intrinsic to cognition and emotional experience. According to Sheets- Johnstone [18], often, scientific frameworks reduce this aspect of movement, isolating emotions and thoughts from bodily actions instead of viewing them as necessary elements for meaningful interactions with the world. This framework essentially suggests that the body is not just the channel the mind uses to communicate but it also possesses an intrinsic intelligence revealed through the ways it interacts within its environment. According to Thalmann [19], behavioral animation involves simulating character behavior, from movement to emotional interactions, making each scene unique. Even basic actions like walking vary based on mood, fatigue, or circumstances, making precise modeling difficult. The challenge for future computer animation lies in accurately replicating human behavior while considering social and individual differences. In educational contexts, integrating embodiment into dynamic visualizations, like animations, enhances comprehension. De Koning and Tabbers [20] suggest that visualizations are more effective when learners physically engage with the depicted movement. Strategies include mimicking gestures, using body metaphors, and physically interacting with animations. By linking cognition to sensory and motor experiences, learners form mental representations that connect abstract concepts to bodily experiences. This approach boosts engagement while reducing cognitive load, making learning more accessible and memorable. Similarly, in animation, a character’s believability improves when its movements are not merely predefined or externally controlled but are instead rooted in a logic of embodiment, where perception and action reinforce one another.

A believable character in animation, regardless of whether it is controlled through keyframing player input, puppeteering, or computer-generated behavior, must exhibit actions that feel naturally triggered by internal thoughts, bodily sensations, and external stimuli. The character’s perception of the world—what it sees, hears, and physically feels—must align with its morphology and movement dynamics [10]. For example, a character rubbing their hands due to cold is an embodiment of both cognition (awareness of the cold) and physical response (friction for warmth). Even when no real-world sensors are involved, the animation must imply how the character perceives and processes sensory information. For example, a large, heavy character should move accordingly—but breaking this expectation (e.g., giving a massive creature delicate, swift movements) can also be an intentional design choice to create contrast. By considering embodied cognition, animation techniques across all types of controls can create characters that do more than move; they experience, react, and exist within their digital worlds in meaningful ways. Ultimately, embodiment in animation is not just about movement accuracy but about creating characters that think, feel, and react in ways that make their actions meaningful within their world. Whether through predefined actions, live performance, or computer-generated behavior, the challenge lies in integrating bodily perception, cognition, and environment to create truly believable animated characters.

## 3. Embodied Character Design

Having defined the concept of embodiment within the context of animating characters, in this section, we proceed with examining the core definitions and types of nonverbal behavior communication towards understanding the different ways of creating character personalities through embodied character design.

While words are important, nonverbal cues—glances, posture, embodied interaction, and more—form the essential building blocks and communication channels of nonverbal behavior, serving as a critical framework for understanding personality and emotional expression.

### 3.1. Nonverbal Behavior and Communication Definitions and Types

Nonverbal communication includes the wide range of signals and actions that occur beyond spoken words. Glances, posture changes, and subtle gestures form the core of nonverbal behavior. Studies show that these cues can be just as important, or even more so, than verbal communication for expressing emotions and intentions. For instance, Mehrabian [21,22] estimated that facial expressions, gestures, and other nonverbal cues convey 93% of people’s feelings and attitudes. Likewise, Birdwhistell [23] argued that verbal communication accounts for no more than 30% of the meaning exchanged in social interactions. Understanding these behaviors can help people to interpret unspoken messages and communicate more effectively. This section defines nonverbal communication and outlines its main types.

#### 3.1.1. Cues vs. Signals

**Figure 2:**
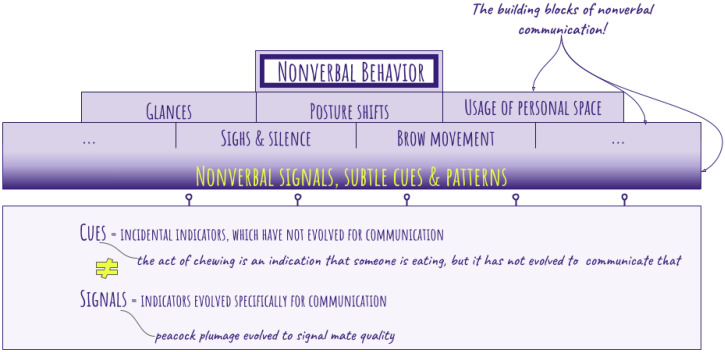
A conceptual schema describing nonverbal communication and behavior.

Before diving into the different types of nonverbal communication and its channels, it would be useful to define the difference between cues and signals. According to evolutionary biologists, cues essentially refer to incidental indicators, which have not, however, evolved for communication. On the other hand, signals have evolved specifically for communication (Figure 2). For instance, the act of chewing is an indication that someone is eating, but it has not evolved to communicate that specifically, while peacock plumage evolved to signal mate quality [24].

#### 3.1.2. Nonverbal Communication Types

Nonverbal communication includes the wide range of signals and actions that occur beyond spoken words. Glances, posture changes, and subtle gestures form the core of nonverbal behavior. Studies show that these cues can be just as important, or even more so, than verbal communication for expressing emotions and intentions. Understanding these behaviors can help people to interpret unspoken messages and communicate more effectively. This section defines nonverbal communication and outlines its main types.

Being the process of sharing information, signals, and messages without using words [25,26] often subtly and unintentionally, nonverbal communication includes various types. Facial expressions involve movements of facial muscles to convey emotional states [27]. Gestures are movements of the hands, arms, or head that express ideas or feelings [28]. Paralanguage refers to vocal elements such as tone, pitch, loudness, and even silence, which provide meaning beyond spoken words [29]. Proxemics focuses on the use of personal space and physical distance in social settings, where personal space acts as the physical area individuals maintain around themselves [30]. Eye gaze, including actions such as looking, staring, or blinking, can reveal emotions and thoughts [31]. Haptics, or communication through touch, conveys various emotions and meanings depending on the context [31]. Body language encompasses physical actions such as posture, gestures, facial expressions, and eye movements, all of which transmit nonverbal messages. Appearance, including clothing, hairstyle, and other personal choices, communicates aspects of mood, personality, and social status [31,32]. Lastly, artifacts—objects, images, or tools such as avatars or icons—represent parts of a person’s identity or personality [31].

Understanding these elements is crucial for effective character design and animation as they allow creators to convey depth, emotion, and intention in a way that resonates with audiences. Characters that exhibit believable nonverbal behaviors can evoke empathy, communicate emotions without dialogue, and create memorable interactions [33]. Thus, the use of nonverbal communication cues in virtual agents can create more persuasive and empathic characters.

For instance, facial expressions and gestures can instantly reveal a character’s emotional state, while body language and proxemics can establish relationships and social dynamics within a scene. Moreover, subtle cues like eye gaze and paralinguistic elements add layers of realism, helping characters to feel alive and relatable. Additionally, choices in appearance and the use of artifacts can enhance storytelling by visually representing a character’s background, personality, or motivations. By incorporating these principles into design and animation, creators can produce more engaging and immersive narratives, leveraging the full spectrum of human communication to connect with their audience. Of course, just like with every creative process, the design of authentic characters requires the use of established theoretical backgrounds and models such as Eckman’s set of basic emotions [34], the Circumplex model of affect [35], or the Five Factor model [36], combined with iterative design and focus groups [37].

## 4. Character Personality as a Response Mechanism

Personality traits can contribute to how characters interpret and react to environmental stimuli, shaping their nonverbal behaviors in distinctive ways. These traits manifest as mechanisms triggered by events occurring in the character’s environmental surroundings, enabling them to sense and respond through the aforementioned nonverbal channels. Thus, personality-driven reactions to stimuli embody a unique combination of communication, interaction, and expression.

One of the most interesting aspects of nonverbal communication research is the relationship between personality-driven expression and the observer’s ability to interpret and embody the sensed data, even when visual appearance is neutralised but morphology (e.g., structure and movement) is preserved. In this section, we examine the different ways personality traits can be interpreted, communicated, or expressed through nonverbal reactions. We also explore what can be communicated through a character’s posture, gestures, and embodied interactions with their environment and others when visual characteristics are minimised. The role of nonverbal behavior as a communicative tool that bridges individual expression and collective interpretation can potentially advance our understanding of embodied interaction beyond surface appearances.

### 4.1. Definitions

Before diving into the ways personality and personality traits act as a stimuli filter and response mechanism, it would be useful to provide some definitions of relevant terms in different scientific fields and disciplines.

In psychology, personality is defined as the enduring characteristics that account for consistent patterns of feeling, thinking, and behaving [38]. Bergner [39] builds upon and refines this definition, describing personality as an enduring set of traits and styles that encompass an individual’s unique natural inclinations and distinctive characteristics within a societal context. When it comes to media, particularly animation, television, and film, personality is conveyed through consistent character traits and behaviors. Thomas and Johnston [6] emphasise how body movements, dialogue, and interactions help to build personality in animated characters, while Hoffner and Cantor [40] and Field [41] similarly argue that physical appearance, speech patterns, and actions allow audiences to infer personality in TV and movie characters. Moving on to the performing arts, Laurel [42] draws a connection to classical dramatic theory, citing Aristotle’s view of characters as “bundles of traits, predispositions, and choices” that come together to form a cohesive entity.

Despite the different fields, all the aforementioned definitions highlight the critical role of portraying characters in a manner that enables viewers to clearly understand their personality. Successfully communicating a character’s personality relies heavily on the audience’s ability to predict their behavior, actions, movements, moods, and attitudes. Ultimately, it is safe to assume that a character’s personality can be effectively conveyed and interpreted when it demonstrates consistent patterns [43].

### 4.2. Personality Traits Through Nonverbal Reactions

In the previous sections, we analyzed patterns from psychology, biology, movement, and the performing arts to explore connections between personality traits, emotions, and nonverbal behavior. These findings were categorised under nonverbal communication types and examined across scientific contexts. In this section, we expand on each nonverbal communication type, identifying and revealing specific patterns and the meanings they potentially convey through a literature review. Regarding nonverbal expressions and connections to personality traits, Mehrabian’s communication model [44,45] highlights that body language and vocal tone often carry more weight than verbal content, particularly during emotionally charged interactions. His formula quantifies this dynamic as follows:

Total Emotion/Attitude Communicated = 7% Verbal + 38% Vocal + 55% Facial

This framework underscores the pivotal role of nonverbal cues in effective communication.

#### 4.2.1. Facial Expressions

**Figure 3:**
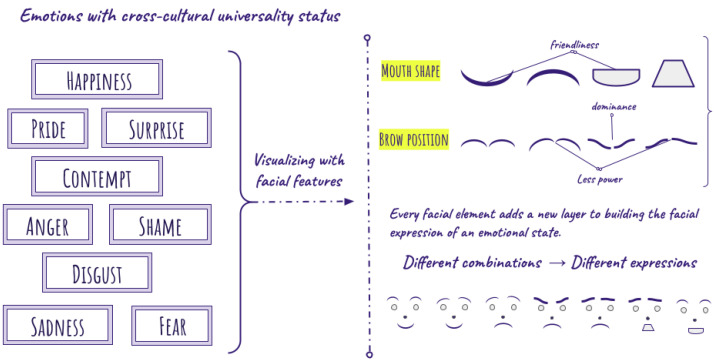
Visualising Ekman’s universal emotions [34] by combining different facial elements to build facial expressions.

Facial expressions are a primary form of nonverbal communication, evolving from survival mechanisms to tools for social interaction. Darwin [46] proposed that expressions serve two functions: preparing organisms for environmental changes and conveying social information. This idea, later expanded into the Two-Stage Model, suggests that facial movements evolved from survival tasks, like rejecting harmful food, to signaling internal states and predicting others’ actions [46,47,48,49]. Ekman [34] identified nine universal emotions, including anger, fear, and happiness, which influence interpersonal trait perception [47]. For example, smiles signal friendliness, while frowns or furrowed brows convey dominance or distress. These facial expressions shape how we communicate emotions and interpret others’ intentions [50,51,52,53]. Consequently, different combinations of expressions of facial features can produce visualizations of different emotional states (Figure 3).

#### 4.2.2. Eye Gaze

Eye gaze plays a dual role in social interaction, allowing individuals to both perceive information from others and transmit it through gaze direction and duration. These factors can convey various messages, including dominance or threat [54,55], attraction [56,57], a need for approval [58,59], or a desire to communicate [60]. The behavior of the transmitter is influenced by their environment, with theories highlighting how social context shapes gaze dynamics and self-presentation.

#### 4.2.3. Body Language → Movement → Position-Posture-Gestures

Body language is a medium that can communicate a great deal of information. However, despite previous beliefs, body language can be quite subtle and indefinite in communicating feelings, moods, and attitudes [31]. In this section, we explore movement, position, posture, and gestures as nonverbal behavior communication channels since they all belong in the realm of body language.

##### Movement Analysis and LMA

Movement analysis involves observing, annotating, and analysing movement, often by certified experts. Among the various frameworks, Laban Movement Analysis (LMA) is one of the most widely used. Developed by Rudolf Laban [61], LMA examines movement through four components: body, effort, space, and shape, collectively forming the BESS system [62,63]. LMA has evolved through contributions from researchers and practitioners [64,65] and is applied across fields, including dance education [66,67], archetypal character and personality creation in dance [68,69], and animation [70]. It remains a key tool for understanding movement’s function, expressivity, and qualities.

##### Movement Qualities

**Figure 4:**
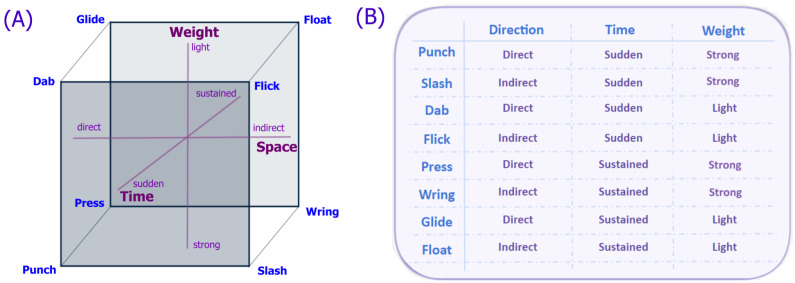
Laban’s Basic Effort Actions. Panel (**A**) visualises Laban’s effort dynamosphere and panel (**B**) lists Laban’s Eight Basic Effort Actions. Source: [69].

Blom et al. [71] define movement qualities as “distinctly observable attributes or characteristics produced by dynamics and made manifest in movement”, describing how bodies move in terms of energy, space, and time. These qualities combine to create unique movement dynamics involving specific values for space, time, forms, or shapes [71,72]. LMA focuses on these qualities within its effort category, analysing motion factors like weight, space/direction, time, and flow—each with polar opposites—resulting in Laban’s eight Basic Effort Actions (Figure 4), linked to both environment and personality traits [62,73]. These efforts have been widely applied in dance [69,74], character design and animation [70], as well as AI and robotics [68,75]. Recent research efforts have proved the effectiveness of the LMA framework to identify the expressed and/or perceived emotion in humans through computer vision [76,77] and AI-driven movement classification [78].

##### Position and Posture

**Figure 5:**
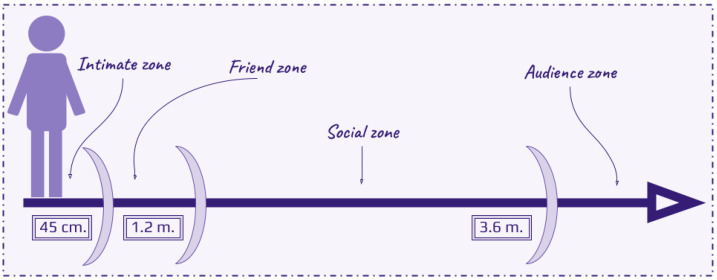
Types of spatial zones for different levels of intimacy, as defined by Dash and Davis [79].

Position and posture are essential elements of nonverbal communication, reflecting personal characteristics and social dynamics. Spatial orientation, or position, illustrates how individuals relate to their environment and others, often categorised by proxemics into zones of intimacy and engagement, as demonstrated in Figure 5: intimate (0–0.45 m), friend (0.45–1.2 m), social (1.2–3.6 m), and audience (beyond 3.6 m) [79]. Frameworks like LMA and Bayesian models help to interpret these spatial behaviors and their psychological implications [80]. Posture also conveys emotions and traits, with upright stances signaling confidence and slouched or bowed positions indicating submissiveness. Friendly traits are often expressed through open body language, forward lean, eye contact, and smiles, sometimes softened by submissive gestures like shoulder shrugs [80]. Together, position and posture play a pivotal role in decoding personality and interactional cues.

#### 4.2.4. Gestures

Gestures, involving movements of the hands, arms, and body, enhance communication by emphasising verbal messages and improving clarity [79]. Rather than focusing on their physical forms, research examines gestures in terms of their movement qualities, which can convey emotional states [81,82]. In 3D character design and animation, recent studies have applied Laban Movement Analysis to develop body movements capable of expressing up to 15 different emotions, demonstrating gestures’ role in nonverbal emotional communication [83].

#### 4.2.5. Paralinguistics

The term paralinguistics encompasses the use of vocal elements like volume, tone, pitch, and speech rate, as well as silence, to communicate without words [29]. Occasionally, paralanguage may include universal verbal signals understood across languages [79]. The Paralinguistics Model of Rapport, introduced by Novick and Gris [84], highlights how nonverbal vocal elements help to establish rapport in conversations. By adjusting pitch, volume, and speech rate, and mirroring others’ vocal traits, the model creates a sense of synchronicity, signals attention, and incorporates social language and humor to build positive connections [84,85].

#### 4.2.6. Haptics

Haptics, or communication through touch, conveys messages of proximity, intimacy, and power dynamics. The type of touch—intimate, formal, or informal—can signal dominance or status, with touch initiation often perceived as asserting power [86,87,88]. Sekerdej et al. [89] identified factors influencing haptic interactions: the initiator, the recipient’s perception, reciprocity, and context. For instance, returning a formal touch, like a handshake, reduces perceived hierarchy, while reciprocating an informal gesture fosters equality in casual settings.

#### 4.2.7. Appearance

Appearance in nonverbal communication reflects self-presentation and can signal mood or personality traits. Clothing, styling, and aesthetic choices serve as tools for expressing or evoking emotional states. According to color psychology, colors and patterns can influence emotions or inspire desired moods, serving as both reflective and motivational tools [31,32,90].

## 5. Sensor-Based Animation

Animation has always been the art of observing, mastering, and recreating human and animal movement as characters’ personality and behavior either by drawing or digitally key-framing. The term sensor-based animation was proposed by Thalman in 1996 [19], suggesting not only the need for the use of physical and virtual sensors, but stressing the importance of predicting the sensorial behavior of the digital character. Over the last few decades, the use of various sensors for capturing movement and human activity has revolutionised the way that digital animation is produced.

In the concept of embodied character design, we suggest that the sensorial system and perception of the character are essential in manifesting this embodied behavior and therefore unraveling its personality. As mentioned in Section 1, there are different types of movement generation and control used for animating characters, including predefined and dynamic approaches. These approaches differ in when and how the character’s bodily behavior is controlled or programmed, and by whom.

Guimmara et al. [91] identify embodiment as a summary of perceptual aspects of the bodily self that include the following aspects:

- Multisensory integration and egocentric frames of reference;
- Proprioception, position sense, and the perception of limb movement;
- Visual capture and visual processing of the human body;
- Motor systems: planning, preparation, and execution of motor schemas.

In the following section, we categorise digital animation based on the sensors that are used not only to create the animation but to simulate the sensorial system of the digital character.

A digital character, depending on the manner of animation, should respond according to their implied sensorial activity as follows:

- Motion Capture and Motion Sensing: Acted sensing by the performer (the actor acts as if they feel or react and their behavior is captured);
- Biosensors: Captured sensing of the performer (actor’s biosignals are captured as indices of what the performer (and therefore the animated character) feels;
- Virtual sensors in Agents: Movement-generation-programmed sensing (agents are programmed to act as they feel a stimulus through AI);
- Physical sensors (morphological computing).

### 5.1. Motion Capture and Motion Sensing

Motion capture and different techniques of motion sensing have been widely used in recent decades in the computer animation, game, and film industries to enhance realism, speed up the animation process, and reduce manual keyframing efforts. These technologies include passive and active optical sensors (e.g., Vicon [92], OptiTrack [93], and Qualisys [94]), inertial sensors (IMU—inertial measurement unit) that provide lower-cost portable suitcases for real-time applications (Xsens [95] and Rokoko [96]), magnetic sensors, as well as pressure and force sensors (e.g., used in foot tracking, facial mocap (for lip syncing), and virtual puppeteering [15]). These technologies are used to capture realistic human motion for films and games, facilitate facial animation, and enhance virtual reality (VR) and augmented reality (AR) experiences. They also support real-time motion capture for immersive applications and enable the animation of non-human entities, such as creature and fantasy characters, based on human movement. Recent developments also explore how we can use collaborative mixed reality environments to enhance motion-capture workflows, allowing performers to interact with digital environments in a more intuitive way [97].

The incorporation of performers in the animation process opens a wide field of possibilities where nonverbal personality traits, communication, and expression are not only designed on screen or paper but embodied through acting, dancing, and performing [98]. On the other hand, acting for the stage or the camera is different from acting for animating a character in many ways.

Subjective perspective and feeling and closed-loop continuous feedback (if in real time) affect how the performer “becomes” the character (moves as the character and nonverbally makes the character express themselves [99].

In this case, the sensorial system of the digital character becomes a hybrid of the performer and the digital avatar as the performer is asked to puppeteer the character but also act as they sense and feel the environment and stimuli that the 3D character senses in this particular part of the scenario [100,101].

### 5.2. Biosensors: Visualising the Inside Out

**Figure 6:**
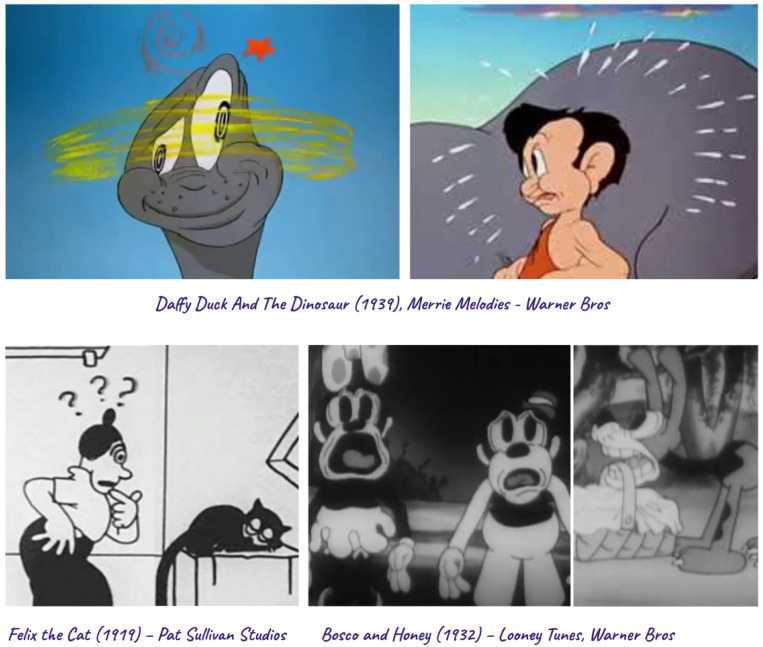
Visualising the inside-out exaggerations of internal functions or feelings in character animations. All examples are from animations in the public domain.

In traditional animations, we have many examples through applying some of the 12 principles of animation [102], e.g., exaggeration, an internal function or feeling of the character is visualised or sonified to convey the feeling. Think of the heart popping out of the body while beating in classic Tom and Jerry [103] to convey the feeling of fear, love, or shock of the character, or exaggerated sweating to show exhaustion, stress, or anxiety. Figure 6 includes some examples of shock, anxiety, confusion, fear, and surprise visualised in animated clips that are currently part of the public domain.

Researchers have shown that expressive biosignals, or biosignals displayed as a social cue, have the potential to facilitate communication as a means to recognise and express our emotions and physical being (Liu et al. [104]). The same work suggests that the incorporation of biosignals in animation has positive effects on easier sharing and social connection. Furthermore, wearable technology is being increasingly integrated into the performing arts to create interactive, responsive environments where physical movements drive digital outputs in real time [105]. Previous work suggests that the incorporation of wearables in psychological research [106] not only helps us to deepen the understanding of felt emotions during different contexts [107,108] but also provides data that can be used both pre-recorded or online to create metaphoric or more literal digital narratives [109,110]. In such cases, the human who provides these internal sensations (heart rate—EEG, electrodrmal activity (EDA), temperature, and electrocardiodiagram (ECG)) through wearable sensors indirectly provides the internal feelings and states of the digital character and can be visualised or sonified in different ways. The use of such sensors in the workflow allows transferring and making visible (or audible) internal sensations of the digital character that are otherwise hidden.

### 5.3. Virtual Sensors in Agents (Autonomous Digital Characters)

The term virtual sensor in animation refers to a software-based technique that simulates the real-world sensor by estimating the motion, physics, or environmental aspects of the digital character. Such sensors can be used as a replacement for motion capture hardware (e.g., Open Pose or Media Pipe) or enhancement of motion capture, in procedural animation, or in AI-driven motion estimation. Virtual sensors in animation refer to software-based data processing techniques that simulate real-world sensor readings without requiring physical hardware. These sensors estimate motion, physics, or environmental conditions using algorithms, AI, and mathematical models. From a technical standpoint, Bayesian Networks have been used to simulate virtual humans, identifying five challenges: uncertainty, controllability for the animator, misinterpretation, interaction (we as humans do not directly react to the stimuli sensed by our sensory organs in an objective manner but, based on the perception, interpretation, and intention, we react according to our personality traits and our internal state (how we feel at this moment or towards the situation or another person), and extensibility.

Other researchers propose the Neural State Machine as a novel data-driven framework to guide characters to achieve goal-driven actions with precise scene interaction [111]. In such cases, the software is programmed to mimic the sensing of the external scene of the virtual character and predict their response. Unlike simulation of the motion itself by systems such as OpenSim (https://simtk.org/projects/opensim, accessed on 18 January 2025) or inverse kinematics models [112], virtual sensors [113] simulate the virtual human behavior, incorporating the behavior model framework that takes into account the sensorial input of the characters (usually vision and its properties such as the field of view), perception, and interpretation based on a personality model. In several examples of simulating the behavior of autonomous actors in a virtual environment or a computer-generated imagery (CGI) scene, one should take into account (a) the particular situation or scenario, e.g., an emergency situation, or meeting a friend with whom we had a quarrel before; (b) the sensorial input of the surrounding environment for each actor, typically relying on calculating the average human vision; and (c) the internal state that is relevant to the emotional and cognitive state of the person/autonomous actor. While in most models the personality is seen as a rule-based model for handling sensorial inputs and reacting accordingly, the autonomous bodily behavior of physical or virtual sensors can leverage a realistic simulation as they can capture more stimuli from the surroundings but also simulate the internal state, bodily sensations, and emotions.

### 5.4. Morphological Computation

One of the most recent trends in digital animation is the notion of morphological computation. The term morphological computation, as defined by Muller [13], describes the principle of computations required for motion execution that are not implemented in a dedicated (electronic) controller but executed by the kinematics and morphology of the body itself. In fact, morphological computation is a field that intersects computer animation, robotics, and sensors [114]. As a field, it explores how the physical body of an agent can influence behavior, learning, and problem solving. Analogous to embodiment in humans, where the embodied mind and the mindful body act as a whole for effective and efficient interaction with the environment, the field of morphological computation suggests that the behavior of the agent is incorporated not only in the software of the agent but in the physical (in the case of a robot) or virtual (in the case of the graphic 3D model) aspects of the character/agent. By “morphological computation”, we mean that certain processes are performed by the body that otherwise would have to be performed by the brain.

## 6. Integrating Sensors in Animation Workflow

While animation is typically about creating or moving an entity that resembles or acts as a living person (or creature in a more general sense) as if it were real or alive, it usually puts the creator in a position of designing through mimicking or imagination. The integration of sensors into digital animation workflows represents a significant shift from traditional keyframing and motion capture techniques towards a more embodied and affective animation paradigm. This approach serves as an opportunity for creators to expand their pool of options outside the realm of their imagination and work with real live data. Wearable technologies and biosensors, including electrocardiogram (ECG), electrodermal activity (EDA), electromyography (EMG), respiration, and skin temperature monitoring, can provide real-time physiological data that can inform character behavior and emotional expression [115,116]. Unlike conventional facial expression tracking or skeletal motion capture, these sensors allow animators to infuse characters with dynamic, context-sensitive responses based on a performer’s internal state. In this section, we explore the different ways sensors and wearables can be integrated in animation workflow, as well as some of the challenges that might arise while following these techniques.

### 6.1. Sensor-Based Emotion Capture in Animation

Traditionally, animation has relied on predefined expressions and keyframe interpolation to depict character emotions. However, the use of biosignals as input for animation systems enables a more organic and adaptive approach. Heart rate variability (HRV) has been identified as a strong indicator of arousal and stress, with reduced HRV often linked to heightened emotional states such as anxiety or excitement [117]. This makes HRV a potential driver for subtle adjustments in a character’s breathing rate, posture, or micro-movements to reflect internal tension or relaxation. Similarly, EDA, which measures sweat gland activity, has been widely used as a marker of emotional arousal and cognitive engagement [118,119]. For instance, Giomi et al. [120] discuss a phenomenological approach to using wearable technology, suggesting that biosignal sonification can provide real-time feedback while also actively reshaping the performer’s bodily awareness and interactions within digital environments. Moreover, recent research in the field of health has demonstrated the potential for motion-tracking technologies to advance healthcare by providing real-time data-driven insights into human movement, offering valuable techniques that could extend to sensor-based animation [121].

In an animation context, such signals and data could be used to modulate real-time shader effects, environmental changes, or secondary animations, such as subtle twitches, muscle tension, or dilation of pupils, to simulate affective responses. Electromyography (EMG), which captures facial and body muscle activity, is another powerful tool for refining expressive animation. Previous studies have demonstrated that facial EMG signals correlate with emotional valence, making them particularly useful for enhancing facial animation beyond predefined morph targets or blend shapes [122]. By incorporating EMG data, digital characters can express more nuanced micro-expressions, such as tension around the mouth during stress or involuntary eye twitches during excitement. This aligns with Paul Ekman’s [123] work on facial action coding, which categorises muscle activations that correspond to universal emotional expressions. By integrating EMG-based muscle activity tracking, animators can create more lifelike facial animations that respond dynamically to the performer’s physiological state.

### 6.2. Contextual and Narrative Integration of Biosignals

Rather than directly mapping biosignals to predefined emotional states, an alternative approach is to use them as narrative modifiers that influence the animation workflow contextually. Damasio’s Somatic Marker Hypothesis [124] highlights how emotions serve as embodied decision-making signals, meaning that characters could be animated in ways that reflect their physiological state rather than simply displaying surface-level expressions. For example, in a suspenseful scene, increased heart rate and shallow respiration [125] could trigger subtle tension in the character’s shoulders, faster blinking, or changes in ambient lighting to create an immersive experience. Lisa Feldman Barrett’s Theory of Constructed Emotion [126] challenges the notion of universal emotional expressions, instead proposing that emotions are constructed based on past experiences and situational interpretation. In an animation pipeline, this perspective suggests that, rather than using rigid emotion classification, biosignal data can be used as input for procedural animation systems that adjust a character’s behavior dynamically based on scene context, character history, and environmental cues. For instance, if a character’s biosignal data suggest increased arousal but neutral valence, the system might infer that they are excited rather than scared, leading to more animated gestures and energetic movement rather than defensive body language.

### 6.3. Challenges and Considerations in Sensor-Driven Animation

While sensor-driven animation offers new possibilities for enhanced expressivity and interactivity, it also presents several challenges:

- Noise and Variability: Biosignals are inherently noisy and sensitive to movement artifacts, making real-time animation challenging [115]. Filtering techniques and machine learning models must be implemented to distinguish genuine emotional signals from environmental interference.
- Individual Differences: Emotional responses and baseline biosignals vary across individuals, meaning that one-size-fits-all emotion models are ineffective [126]. Instead, personalised calibration may be required to adapt the animation system to each performer’s unique physiological patterns.
- Interpretation Complexity: Unlike direct motion capture, biosignals do not correspond to explicit movements or expressions, making their integration into animation workflows less straightforward. Instead of focusing on emotion detection, the emphasis should be on using biosignals as modulation parameters that influence motion curves, shaders, and secondary animations dynamically.

Despite these challenges, the potential for sensor-enhanced animation workflows is vast. By integrating wearable biosensors with motion capture, digital characters can be animated not only with physical realism but also with affective depth, paving the way for more immersive interactive storytelling, virtual performances, and emotionally responsive avatars in gaming, VR, and cinematic animation.

## 7. Proposed Workflow and Discussion

In the previous sections, we provided an extensive literature review, exploring the concept of embodiment through the lenses of multiple different fields. Driven by the insights gathered, in this section, we present our proposed workflow for integrating sensors within the process of digital animation.

### 7.1. Methodology: A Sensor-Based Approach to Animation

**Figure 7:**
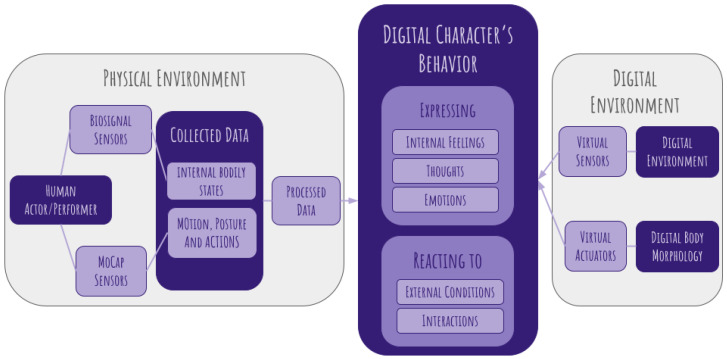
Model of our proposed workflow for an embodied perspective in digital animation.

Our proposed workflow (Figure 7) expands on the initial schema pictured in Figure 1, integrating multiple sensor types to capture both internal bodily states and external motion dynamics from human performers. This approach leverages biosignal sensors (such as EMG, ECG, and EDA) to track physiological changes and motion capture (MoCap) sensors to record body movements, posture, and facial expressions, as discussed in Section 6. These collected data streams undergo processing and are mapped to digital characters, enabling them to express emotions and respond dynamically to virtual interactions. The ultimate goal is to create a feedback-driven animation pipeline that enhances realism, emotional depth, and user engagement.

As pictured in Figure 7, the proposed pipeline integrates multiple types of sensors into the digital animation process, structured as follows:

- Physical Environment: A human performer is equipped with biosignal sensors (e.g., EMG, HRV, and GSR) and motion capture sensors through wearables to track both internal bodily states and external movements. This combination allows for the real-time extraction of physiological and kinematic data, which are processed and used to create the digital character’s behavior.
- Digital Character Integration: The collected data inform digital character behavior:
  – Characters express internal states such as emotions and thoughts based on biosignal data.
  – Characters react to external conditions, dynamically adjusting responses within the space of the digital environment.
- Digital Environment Interaction: Virtual sensors and actuators allow the character to perceive and modify its digital surroundings. The digital body morphology adapts based on sensor inputs, ensuring a cohesive interaction between the character and its environment.

This workflow also considers the practical implications for animators, offering tools for integrating sensor data into character animation pipelines with minimal manual intervention. By automating aspects of movement and expression generation, animators can focus on refining high-level stylistic and narrative elements rather than manually keyframing each motion.

### 7.2. Discussion: Implications and Challenges

The proposed workflow showcases the potential to advance sensor-based animation through multimodality. It is important, however, to recognise and voice possible challenges affecting its implementation and scalability. Firstly, biosignals are inherently noisy and sensitive to movement, making real-time animation a challenging task [115]. Especially when it comes to combining multimodal sensor data, possible synchronization issues between biosignal and motion capture data require advanced filtering and machine learning techniques to ensure accurate mapping to digital characters. In such cases, robust signal processing pipelines must be implemented to differentiate and separate genuine emotional signals from environmental interference to avoid the risk of generating false animations.

As discussed in Section 6.3, another key challenge lies in the inability of generic models to capture the unique physiological patterns of each performer since emotional responses and baseline biosignals can vary across individuals [126]. Additionally, since biosignals do not directly translate into clear movements, their use in animation can be quite complex, hence making their use more appropriate for subtly adjusting motion and animations rather than relying solely on them for production. Furthermore, biosignal-driven systems can increase cognitive load and affect natural movement patterns. While feedback enhances immersion, too much reliance on it may reduce spontaneity and creativity. Therefore, finding the right balance between user control and system automation is essential for effectively integrating biosignals into animation.

## 8. Conclusions

In this paper, we introduce the concept of embodied character design for digital characters and agents, emphasising how various sensors can enhance their responsiveness. These sensors may capture human movement and biosignals or be programmed to help agents perceive their internal and external environments more effectively. We differentiate types of computer animation based on their use of virtual and physical sensors and explore how animations are controlled—whether by human input, coding, or AI-driven agents.

The idea of embodied character design and sensor-based animation necessitates a highly interdisciplinary approach, spanning computer graphics, artificial intelligence, and robotics, as well as the performing arts, traditional character design, and the study of embodiment through psychology, neuroscience, and cognition. A significant portion of this paper is dedicated to examining embodied interaction, nonverbal personality traits, and communication cues—frameworks that enable scientists and artists to create digital characters or agents with enriched embodied personalities.

By framing personality as a mechanism, we argue that it emerges as a response to internal and external stimuli. Therefore, expanding a character’s sensory capabilities enhances both its personality and believability. A digital character—whether human or non-human—is animated through virtual, physical, or implied interactions with these stimuli.

The contributions of this work can be summarised in the following points: (a) driven by the recent advancements in the literature, we proposed an animation model workflow that incorporates both physical and virtual sensors to capture the nonverbal cues of digital characters. While human actors’ and performers’ movements, facial expressions, and biosignals can be captured through motion capture technologies and wearables, virtual sensors enabled by AI and procedural animation can be used to activate the characters’ changes in action and morphology when sensing objects or cues in their virtual environment; (b) we presented the recent advancements in the technological and analytical tools that can potentially be applied in combination to create empathic and believable characters; (c) last but not least, we highlighted the importance of the simulated or acted sensorial reactions of the character to unravel their personality through nonverbal behavior. The framework that is presented and discussed in this work aims to serve as a theoretical tool for future designers. Summarising the recent advancements and applications of the framework that connect the nonverbal behavior of the character that is enabled or controlled through biosignals is part of our future work.

As part of the IMAGINE MOCAP project, from which this research emerges, we aim to further explore how motion capture sensors, wearables, and live coding contribute to the development of digital narratives in animation, games, interactive media, and transmedia networks. By integrating engineering and human–computer interaction with the practical and theoretical frameworks of the performing arts—such as puppeteering, acting, and dance—we seek to advance the concept of embodied character design.

## Data Availability

The original contributions presented in this study are included in the article. Further inquiries can be directed to the corresponding author.

## Abbreviations

The following abbreviations are used in this manuscript:

AR	Augmented Reality
BESS	Body Effort Shape System
CGI	Computer-generated Imagery
ECG	Electrocardiogram
LMA	Laban Movement Analysis
VR	Virtual Reality
